# Eicosanoid Control Over Antigen Presenting Cells in Asthma

**DOI:** 10.3389/fimmu.2018.02006

**Published:** 2018-09-04

**Authors:** Nincy Debeuf, Bart N. Lambrecht

**Affiliations:** ^1^Laboratory of Immunoregulation, VIB-UGent Center for Inflammation Research, Ghent, Belgium; ^2^Department of Internal Medicine, Ghent University, Ghent, Belgium; ^3^Department of Pulmonary Medicine, Erasmus Medical Center, Rotterdam, Netherlands

**Keywords:** eicosanoids, prostaglandins, leukotrienes, asthma, dendritic cells, macrophages

## Abstract

Asthma is a common lung disease affecting 300 million people worldwide. Allergic asthma is recognized as a prototypical Th2 disorder, orchestrated by an aberrant adaptive CD4+ T helper (Th2/Th17) cell immune response against airborne allergens, that leads to eosinophilic inflammation, reversible bronchoconstriction, and mucus overproduction. Other forms of asthma are controlled by an eosinophil-rich innate ILC2 response driven by epithelial damage, whereas in some patients with more neutrophilia, the disease is driven by Th17 cells. Dendritic cells (DCs) and macrophages are crucial regulators of type 2 immunity in asthma. Numerous lipid mediators including the eicosanoids prostaglandins and leukotrienes influence key functions of these cells, leading to either pro- or anti-inflammatory effects on disease outcome. In this review, we will discuss how eicosanoids affect the functions of DCs and macrophages in the asthmatic lung and how this leads to aberrant T cell differentiation that causes disease.

## Primer on eicosanoids, prostaglandins and leukotrienes

**Figure 1:**
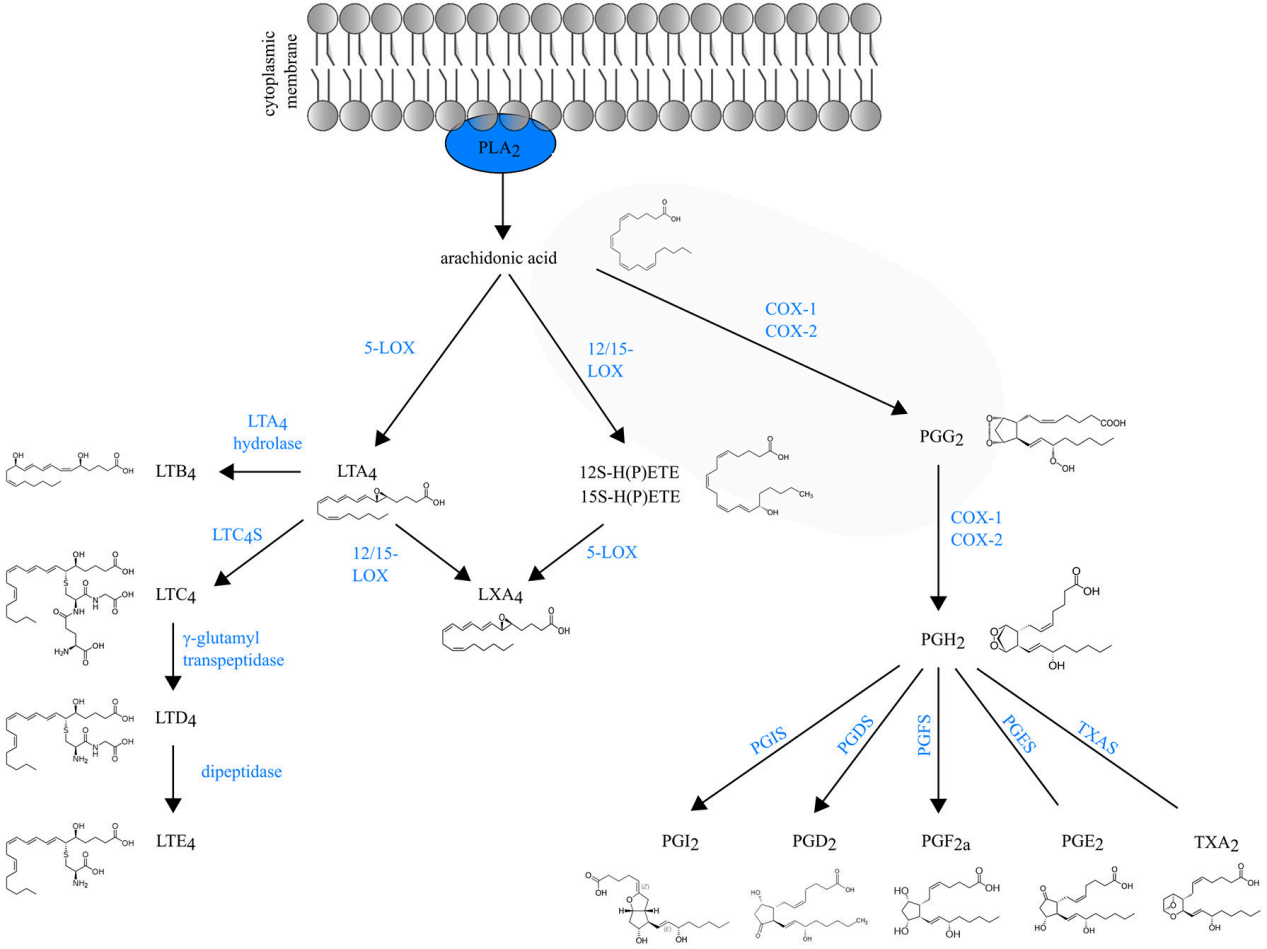
Schematic overview of eicosanoid biosynthesis. Arachidonic acid that is released from membrane phospholipids by cytosolic phospholipase A2 (PLA_2_), can be enzymatically converted either to prostaglandins (PG) and thromboxane (TXA_2_) by COX enzymes or to LT and lipoxins (LXA_4_) by lipoxygenases (LOXs).

Eicosanoids are an important class of biologically active molecules, comprising prostanoids, leukotrienes (LTs) and lipoxins that have important pro- and anti-inflammatory effects in asthma. Under a variety of non-specific activation stimuli, such as pro-inflammatory mediators and other stress, the precursor molecule arachidonic acid (AA) is released from membrane phospholipids by cytosolic phospholipase A2. AA can be enzymatically converted either to prostanoids [prostaglandin (PG) and thromboxane] by COX enzymes or to LT and lipoxins by lipoxygenases (LOXs) (Figure 1).

*Prostanoids* The COX isozymes (constitutive COX-1 and inducible COX-2) catalyze the formation of PGG_2_, which is then reduced to the intermediate PGH_2_ through peroxidase activity. Various cell-specific PG synthases convert PGH_2_ to biologically active products, such as PGE_2_, PGI_2_, PGD_2_ and PGF_2a_ and thromboxane (TXA_2_) (1). The differential expression and the distribution of these enzymes within cells present at sites of inflammation will determine the profile of prostanoid production. For instance, mast cells predominantly generate PGD_2_ through their expression of hematopoietic PGD synthase (hPGDS). Through microsomal PGE_2_ synthase (mPGES-1), PGE_2_ is produced by virtually all lung cell types, but the most abundant sources are epithelial cells, fibroblasts, and macrophages (1). Prostanoids act in both paracrine and autocrine fashion through G protein-coupled receptors (GPCRs) on the surface of target cells. Interestingly, the distribution of prostanoid receptors on immune cells differs from the distribution of prostanoid-specific synthases. Prostanoid synthases are mainly expressed on innate immune cells, whereas prostanoid receptors are expressed on both innate and adaptive immune system leukocytes (2). So, during inflammation, activated innate immune cells will produce prostanoids that act on lymphocytes in a paracrine manner and also modulate their own function in an autocrine way (3).

*Leukotrienes* are generated by LOX enzymes. The different LOX enzymes are named based on their positional specificity of AA oxygenation. For instance, 12-LOX oxygenates AA at carbon 12, resulting in 12-hydro(peroxy)eicosatetraenoic acid [12-H(P)ETE] (4). Since the human leukocyte-type 12-LOX is very similar to reticulocyte-type 15-LOX, these enzymes are often referred to in the literature as 12/15-LOXs (5). Furthermore, mice do not express 15-LOX and only express the leukocyte-derived 12-LOX. Because murine 12-LOX is also able to generate 15-H(P)ETE, the enzyme is often designated as 12/15 LOX as well (6).

5-lipoxygenase (5-LOX) generates the leukotriene LTA_4_, an unstable intermediate, which is converted to the chemoattractant LTB_4_ or to nonchemotactic LTC_4_ by the cytosolic LTA_4_ hydrolase enzyme or leukotriene C4 synthase (LTC_4_S) respectively. LTC_4_ is exported to the extracellular space and is further converted to the unstable LTD_4_ and subsequently to the stable end-metabolite LTE_4_ (7). LTC_4_, LTD_4_ and LTE_4_ belong to the so-called cysteinyl leukotrienes, due to the presence of the amino acid cysteine in their structure. There are at least three different cysteinyl leukotriene receptors (CysLTR1, CysLTR2, and CysLTR3). LTE_4_ preferably binds CysLTR3 (8), whereas LTC_4_ binds CysLTR2 and LTD_4_ binds both CysLTR1 and CysLTR2 (9, 10).

Leukotrienes are predominantly produced by leukocytes, hence their name. However, the specific profile of LTs produced depends on the cell type. Neutrophils produce exclusively LTB_4_, whereas mast cells, basophils and eosinophils mainly produce cysLTs. Macrophages and DCs synthesize both LTB_4_ and cysLTs (11).

*Lipoxins* (LXA_4_ and LXB_4_) are short-lived eicosanoids that are derived from arachidonic acid through sequential activity of 5-LOX and 12/15-LOX. 15-LOX is a key enzyme for lipoxin generation in the human lung and is expressed by many cells during inflammation, including macrophages, eosinophils and bronchial epithelial cells (12–14).

## Eicosanoids have multiple effects in allergic asthma

**Figure 2:**
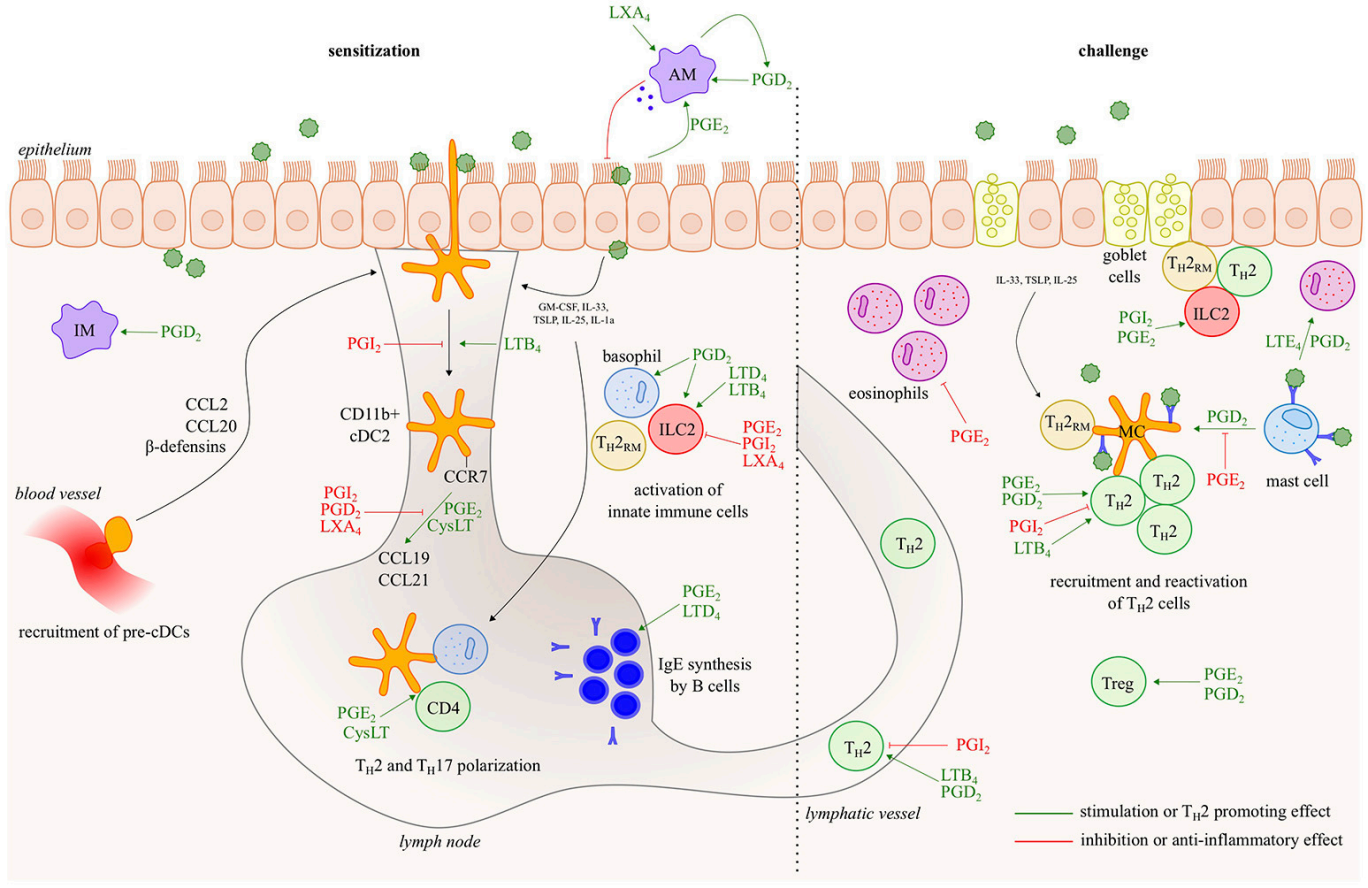
Eicosanoids have multiple effects in allergic asthma. In response to allergens and/or instructive cytokines by epithelial cells and innate immune cells, dendritic cells activate Th2 immunity in the draining lymph nodes, leading to IgE responses and to Th2 effector cells that control eosinophilic airway inflammation, goblet cell metaplasia and bronchial hyperreactivity upon return to the lung. Virtually all cell types relevant to Th2 pathology such as Th2 cells, ILC2s, mast cells, basophils and epithelial cells, generate leukotriene/prostaglandin mediators, and/or express receptors for those eicosanoids. The figure represents a schematic overview of eicosanoid functions described in this review.

Asthma is a chronic inflammatory disease of the airways, characterized by reversible bronchoconstriction, airway remodeling and mucus production. Most childhood-onset asthma and half of the adult-onset asthma cases are allergic, identified by a positive skin prick test or the detection of serum IgE antibodies against common antigens, such as plant and tree pollen, animal dander, house dust mites (HDM) and fungal spores. Virtually all cell types relevant to Th2 pathology such as Th2 cells, ILC2s, mast cells, basophils, epithelial cells, smooth muscle cells and fibroblasts generate LT and/or PG mediators, and/or express receptors for those eicosanoids (Figure 2). Among prostanoids, PGD_2_ released from mast cells, has long been implicated in allergic diseases (15). PGD_2_ is known to have chemotactic effects on eosinophils, basophils, Th2 lymphocytes and ILC2s acting via the DP2/CRTh2 receptor (16, 17) and in this way contributes to airway hyperresponsiveness, IgE and cytokine secretion (18–20). PGD_2_ levels and the number of CRTH2+ cells are increased in bronchoalveolar lavage (BAL) fluids from severe asthmatics compared to those with milder disease (21). Several CRTH2 antagonists have shown encouraging results in clinical trials for asthma, further supporting for the role of PGD_2_ in allergic diseases and its potential as a therapeutic target (22).

Although cyclooxygenase and its products, PGs, have been traditionally linked to all four cardinal signs of inflammation (redness, swelling, heat, pain), prostanoids may also have an inhibitory role on inflammatory cells. This discrepancy can be explained by the fact that typical inflammation hallmarks are the result of actions on microvasculature, hypothalamus and nerves, rather than on immune cells. In mouse models of asthma, PGs have pleiotropic effects. PGI_2_ can abolish asthma development by inhibition of DC activation and Th2 cell migration (23–25), whereas PGE_2_ can reduce mast cell secretory responses (26–29) and chemotaxis of eosinophils (30). Furthermore, both PGI_2_ and PGE_2_ can inhibit cytokine release of both Th1 and Th2 CD4 T cells and macrophages (31, 32). Treg differentiation and function is also promoted by PGE_2_ (33, 34).

Prostanoids are also able to inhibit airway remodeling and mucus secretion in asthma models *in vivo* (35–37). It has been shown that PGE_2_ induces fibroblast apoptosis (38), abolishes myofibroblast differentiation (39) and inhibits proliferation of airway smooth muscle cells (40).

In asthma patients, inhalation of exogenous PGE_2_ or its analogs results in bronchodilatation and protection against early- and late-phase bronchoconstriction induced by various asthma triggers (41–43). Despite the benefits of inhaled PGE_2_, it has also been reported that prostanoids can induce irritancy of the upper airway resulting in a reflex cough. However, this can be overcome by treatment with a receptor-selective agonist, as cough is exclusively mediated via the EP3 receptor (44).

In contrary to the bronchodilatory properties of the prostaglandin PGE_2_, CysLTs are an important cause of allergen-induced bronchoconstriction (45). Indeed, treatment with Cysteinyl LT receptor 1 antagonists (LTRAs) attenuates allergen-induced increases in airway hyperresponsiveness (46, 47). Furthermore LTRAs partially attenuate allergen-induced airway eosinophilia (47, 48), demonstrating a more extensive role for LTs in asthma. Indeed, CysLTs that are also released from mast cells, particularly LTE_4_, can cause eosinophil chemotaxis in allergic asthmatics (49). Interestingly, CysLT levels are also increased in BAL fluid (50) and in urine after allergen challenge (51). Currently, LTRAs (such as montelukast) are clinically available. Although these drugs are superior to placebo at decreasing asthmatic symptoms and exacerbations, LTRAs are not recommended as first line therapy for asthma. The reason for this is that they are generally inferior to inhaled corticosteroids in anti-inflammatory and clinical effects^1^. Furthermore, about one third of the asthma patients does not respond to LTRAs (52).

Another type of leukotriene, LTB_4_, through its actions on the BLT1 receptor, is an activator and chemoattractant for different cell types such as T cells (53, 54) and DCs (55). OVA-induced allergic inflammation was completely abolished in BLT1 deficient mice, demonstrating the importance of BLT1 and its ligand LTB_4_ in the development of allergic airway inflammation (56).

Lipoxins have a pro-resolution role in allergic airway inflammation. In severe asthmatics, blood LXA_4_ levels and leukocyte LXA_4_ generation are reduced compared to those with milder disease (57–59). In a mouse model of asthma, administration of a stable analog of LXA_4_ resulted in a diminished airway hyperresponsiveness and pulmonary inflammation (60, 61). Similar results were obtained with resolvins and protectins. Those mediators are also generated by LOX enzymes, but are derived from omega-3 polyunsaturated fatty acids instead of the substrate arachidonic acid (62–64).

Finally, absence of all eicosanoids impairs the induction of a Th2 response and reduces airway inflammation. This has been shown with mice lacking group V secretory phospholipase A2 (sPLA2), which is the enzyme that releases AA from membrane lipids and catalyzes the first step of eicosanoid generation. Deletion of sPLA2 attenuates cell migration and airway hyperresponsiveness, whereas sPLA2 overexpression is associated with severe asthma (65–68). An impaired antigen capture activity and maturation of DCs is responsible for the inhibition of asthma development in sPLA2^−/−^ mice (69).

## Current insights in allergic asthma pathogenesis: A central role for dendritic cells

In allergic asthma, airway DCs take up allergens across the epithelial barrier and subsequently activate Th2 immunity in the draining lymph nodes, leading to IgE responses and to Th2 effector cells that control eosinophilic airway inflammation, goblet cell metaplasia and bronchial hyperreactivity upon return to the lung (70, 71). The central role for DCs in the development of allergic asthma has been demonstrated in numerous studies. Adoptive transfer of GM-CSF-cultured bone marrow-derived DCs (BMDCs) or splenic DCs that were pulsed with ovalbumin (OVA) antigen *in vitro* can sensitize mice, leading to a Th2 response and eosinophilic inflammation upon challenges with OVA aerosol (72, 73). Likewise, DCs originating from the lungs of allergen-exposed mice are also able to induce sensitization when transferred to naive recipients (74, 75). This holds also true for chronic asthma models as repeated DC injection into the lung induces irreversible airway remodeling, characterized by subepithelial collagen deposition and increased peribronchial airway smooth muscle volume (76).

In addition to these studies demonstrating that DCs are sufficient for induction of Th2 immunity in the lung, DCs are also required for inducing a Th2 response to allergens, even in very young mice before weaning (77). Depletion of lung DCs in CD11c-DTR transgenic mice during the first exposure to the inhaled HDM allergen impeded the development of lung eosinophilia and Th2 cytokine production (74). Likewise, DCs are also required for optimal Th2 immunity against other allergens, such as papain and helminths (78, 79).

Beside the crucial role of DCs in inducing Th2 immunity in naïve animals, DCs have also a non-redundant role during the secondary immune response (76, 80). During the challenge phase, DCs are closely located to antigen-specific T cells around the airways and large blood vessels (81). Here, they might secrete chemokines to attract effector T cells or they might restimulate resident memory T cells by providing costimulatory molecules (75, 82).

Murine lungs in steady state contain three major subsets of DCs with specific phenotype and functions; pDCs, IRF8-dependent XCR1+ CD103+ cDC1s and IRF4-dependent CD11b+ SIRPα+ cDC2s. However, during inflammation monocyte-derived DCs (MCs) emerge, coming from monocytes that migrate to the local tissue and upregulate the expression of CD11c and MHC-II (75, 83). They can be distinguished from CD11b+ cDCs by the expression of the Fc receptors CD64 and MAR1 (75). Various studies have shown that CD11b+ cDC2s are the responsible DC subtype for Th2/17 induction upon allergen challenge (75, 84–86). MCs rather play a role during the effector of the immune response, by interacting with effector Th2 cells that migrate back to the lung or with resident-memory T cells (87). In contrast to CD11b+ cDC2s, CD103+ cDC1s play a redundant role in the HDM-driven asthma model (75). There is even literature suggesting that cDC1s induce a tolerogenic response to inhaled allergens (88–90). An immunoregulatory role has also been described for pDCs. Indeed, it has been shown that pDCs in the lung are essential to induce inhalation tolerance to harmless antigens like OVA (91, 92). Furthermore, depletion of pDCs during sensitization or challenge to OVA or HDM allergen might exacerbate inflammation, as immunoregulatory regulatory T cells fail to function properly in the absence of pDCs (91, 93, 94).

Although DCs express PRRs and can sense the environment directly, the epithelium has been shown to be equally important in activating DCs in response to allergens (95). As this is beyond the scope of this review, we refer to Hammad and Lambrecht for a recent review describing the role of epithelial cytokines in the activation of DCs during allergic inflammation (96). In brief, DCs get activated by epithelial cytokines like IL-33, GM-CSF, IL-1a, IL-25, and thymic stromal lymphopoietin (TSLP). The same cytokines also activate ILC2s, basophils and Th2 effector cells to become cytokine producing cells and contribute to the initiation of a Th2 response (70, 71, 97). The release of epithelial cytokines is elicited by environmental stimuli of asthma, such as HDM, viruses, diesel particles and cigarette smoke. On the other hand, protective environments, such as farm dust or lipopolysaccharide exposure, have the potential to suppress this cytokine release and DC activation (98).

## Eicosanoids affect the migration of dendritic cells

The control of DC migration is pivotal for the initiation of cellular immune responses. Upon activation by inflammatory stimuli, DCs upregulate the chemokine receptor CCR7 and home to lymphoid organs, where the CCR7 ligands CCL19 and CCL21 are expressed. This migratory capacity of DCs requires environmental instruction by PGE_2_. PGE_2_ has no effect on the expression of CCR7 on DCs, but couples CCR7 expression to signal transduction pathways such as activation of cAMP-dependent protein kinase A (PKA) and Rho Kinase (99). These signals allow the DCs to start migration, among other by inducing a rapid disassembly of podosomes (100). Surprisingly, PGE_2_ was only required at early time points of maturation to enable DC chemotaxis, whereas PGE_2_ addition has no effect during terminal maturation. Mouse DCs exclusively rely on EP4 receptor triggering for migration, whereas human MCs require a signal mediated by EP2 or EP4 either alone or in combination (101, 102).

In contrary to PGE_2_, PGD_2_, and PGI_2_ inhibit the maturation and migration of DCs. In the skin, Angeli and colleagues showed that parasite-derived PGD_2_ inhibits the migration of epidermal Langerhans cells to the skin draining lymph nodes and affects the subsequent cutaneous inflammatory reaction (103). Similarly, intratracheal instillation of FITC-OVA together with PGD_2_ inhibits the migration of FITC+ lung DC to draining LNs. Activation of the DP1 receptor was responsible for this inhibition (104). DP1 activation also lowers the expression of costimulatory molecules on DCs and enhances the induction of Foxp3+ Treg cells, resulting in an abolished asthma phenotype (34). Inhalation of iloprost, a stable PGI_2_ analog, also suppressed the cardinal features of asthma by interfering with the function of lung myeloid DC. Furthermore, iloprost-treated DCs no longer induced Th2 differentiation from naive T cells or boosted effector cytokine production in primed Th2 cells, showing that the effect of iloprost was DC intrinsic (23).

CysLT enhance the migration of DCs. Indeed, DCs lacking the LTC4 transporter multidrug resistance-associated protein 1 (MRP1) failed to migrate to the lymph nodes, whereas exogenous LTC_4_ or LTD_4_ could restore this migration. However, these CysLTs only promoted optimal chemotaxis to the chemokine CCL19, but not to other related chemokines (105). On the other hand, lipoxins were able to inhibit DC migration (106).

## Eicosanoid signaling in DCs modulates instruction of T cell differentiation

Upon DC-T cell encounter, DCs produce cytokines that drive Th differentiation. The secretion pattern of these cytokines, and thus the Th1/Th2 balance can be modulated by a variety of biologically active mediators synthesized by innate and adaptive immune cells. Eicosanoids such as PGE_2_ exert a great impact on this regulation. For instance, the ratio of PGE_2_ and IL-12, both produced by APCs, may control the balance between Th1 and Th2 immunity (107). Basically, it has been shown that PGE_2_ is a potent inhibitor of IL-12 production (108) and in this way favors a Th2 response (109–112). PGE_2_ also inhibits the secretion of TNF-α from murine DCs (113, 114). The inhibitory role of PGE_2_ on DC cytokine secretion can also be indirect by inducing IL-10 secretion (108, 113, 115). Due to its inhibitory effect on IL-12, PGE_2_ also indirectly inhibits IFN-γ secretion by T cells and NK cells (110, 116).

Beside the Th2 inducing role for PGE_2_, it has also been reported that PGE_2_-treated DCs can induce Th1 and Th17 responses. Adding PGE_2_ together with TNF-α to human BMDCs stimulates IL-12 production by DCs, favoring a Th1 response (117–121). PGE_2_ also stimulates IL-23 production by cultured BMDCs and promotes in this way Th17 differentiation (122, 123).

Prostanoids can also directly modify production of Th cytokines from polarized T cells. PGE_2_ can favor Th2 immunity by inhibiting IL-2 and IFN-γ production by Th1 cells, but not the production of IL-4 by Th2 cells (124, 125). However, in a mouse model of asthma, PGE_2_ has also been shown to inhibit Th2 responses via direct effects on the EP2 receptor on T cells (126). Furthermore, PGE_2_ regulates Th17 cell differentiation and cytokine secretion directly through EP2/EP4 receptor signaling on T cells (127). Via DP1, PGD_2_ can block the expression of the Th1 cytokine IFN-γ. Furthermore, Th2 cytokine secretion is increased through CRTH2 signaling (128). On the other hand, PGI_2_ can directly inhibit production of Th2 cytokines from Th2 polarized mouse splenic CD4+ cells (32, 129), thus directly exhibiting lower levels of Th2 response.

Less is known about the role of leukotrienes on T cell polarization. Machida *et al*. reported that *in vitro* treatment with LTRAs modifies the cytokine profile of DCs (130). By *in vivo* administration of LTRAs, Okunishi and colleagues showed that LTs promote DC antigen presentation and both Th1 and Th2 polarizing cytokine secretion (131).

Furthermore, using ${\text{LTC}}_{4}^{-/-}$ and CysLTR1^−/−^ mice, it has been demonstrated that leukotrienes are crucial for the initiation of a Th2 response upon HDM-dependent Dectin-2 activation on DCs (132). Through CysLTR1, LTD_4_ can induce IL-4 secretion by ILC2s, contributing to Th2 polarization as well (133). CysLTR1 can also be up-regulated in activated CD4+ T cells themselves and can mediate their chemotaxis to LTD_4_, but whether cysLTs exert a direct effect on cytokine production by CD4+ T cells remains unclear (134). This is different from the leukotriene LTB4, which increases cytokine production by T cells (135), but does not affect antigen presentation and cytokine production by DCs (55). Strikingly, mice deficient in CysLTR2 or adoptive transfer of DCs lacking CysLTR2 developed markedly enhanced Th2 immunity to HDM. In fact, CysLTR2 negatively regulates cell surface expression and receptor signaling of DCs (136). Thus, the biologic activity of CysLTs can be tightly regulated by competition between the different expressed CysLT receptors.

12/15-LOX enzymes, required for lipoxin synthesis, are also involved in the modulation of Th2 cytokine secretion. In response to IL-13, DCs secrete the lectin Ym1/2 that might interact with 12/15-LOX in or at the surface of T cells. 12/15-LOX generates 12-HETE that has been shown to reduce Th2 cytokine secretion both *in vivo* and *in vitro*. Furthermore, 12-HETE attenuated airway eosinophilia in an OVA-induced allergic asthma model. However, DC-secreted Ym1/2 was able to decrease the expression of 12-HETE, suggesting that the asthma-promoting effects of Ym1/2 might be explained by inhibiting 12/15-LOX on T cells (137).

## Eicosanoids also affect antigen-presenting B cells and ILC2s

The most described function of B cells is their production of antigen-specific immunoglobulins. However, in addition to antibody production, activated B cells also play a role as accessory antigen-presenting cells. Although they are not as potent as DCs in priming naïve T cells, they are abundantly present in T-cell inductive sites, express costimulatory molecules and produce cytokines that activate DCs and naïve T cells (138–141). Their antigen-presenting and Th2-promoting effects have also been demonstrated in murine asthma models, with a particular role during secondary challenge and when the antigen dose is limiting (142).

Numerous studies have shown that eicosanoids are required for both the development and function of B lymphocytes (143–147). PGE_2_ is necessary for IgE production both *in vitro* and *in vivo*, by affecting IgE class switching (145, 148–151). Furthermore, PGE_2_ has been demonstrated to regulate B cell proliferation (152). Interestingly, PGE_2_ is also able to lower MHCII expression on B cells (148), but whether this affects antigen presentation is still unclear. Leukotrienes, in particular LTD_4_, can enhance immunoglobulin production as well (153). In contrast, lipoxins have the opposite effect as 12/15-LOX deficiency protects mice from allergic airway inflammation by increasing secretory IgA levels (147).

Strikingly, ILC2s have also been shown to present antigen (154, 155). In response to the parasitic worm *Nippostrongylus brasiliensis*, MHC class II expression on ILC2s was required for the induction of an efficient Th2 response. ILC2s express the costimulatory receptors CD80 and CD86, acquire and process antigen and interact with antigen-specific T cells. During this interaction, T cell-derived IL-2 promotes ILC2 proliferation and IL-13 production (155) and this can be affected by eicosanoids. PGD_2_ and CysLTs stimulate Th2 cytokine production from ILC2s (133, 156, 157), whereas other lipid mediators have suppressive roles on ILC2 function. The pro-resolving mediator LXA_4_ could inhibit ILC2 activation (156) and both PGE_2_ and PGI_2_ were able to attenuate ILC2 proliferation, Th2 cytokine generation and resulting type 2 immune response (158, 159).

## Eicosanoids modulate the tolerogenic role of macrophages in the allergic lung

Lung macrophages can be divided into alveolar macrophages (AMs) and interstitial macrophages (IMs). AMs are most abundantly present and are situated in the alveolar lumen, while IMs are located inside the lung interstitium. During inflammation, a third population emerges, as monocyte-derived macrophages infiltrate the alveolar and interstitial areas. Macrophages express different eicosanoid receptors, such as the receptors for PGE_2_ and PGD_2_. Furthermore, macrophages produce both prostaglandins and leukotrienes themselves, allowing autocrine regulation (160).

AMs are sessile, long-lived, and self-renewing cells that derive from fetal monocytes under the influence of GM-CSF (161–164). Several studies have clearly demonstrated that resident AMs induce a tolerogenic response to inhaled antigens (164–171). Use of liposomal clodronate to deplete resident AMs in an OVA or HDM-induced asthma model, favored a Th2 response and subsequently resulted in increased BAL eosinophilia and inflammatory cytokine levels (167, 168). One possible mechanism for this inhibitory role of macrophages is the secretion of SOCS1 and SOCS3 in exosomes and microparticles. The uptake of these particles by alveolar epithelial cells inhibits their activation in a JAK/STAT-dependent way (169). PGE_2_ is a major epithelium-derived factor mediating SOCS secretion (170) and in this way inhibiting the development of allergic lung inflammation (171). Indeed, in a HDM-dependent asthma model, adoptive transfer of PGE_2_-treated macrophages led to a reduction in eosinophilia in the allergic lung (171).

Pulmonary inflammation was also reduced if macrophages lacked group V sPLA2, which is the enzyme releasing AA from membrane lipids and is required for both PG and LT synthesis (172). Those macrophages generated less PGE_2_, resulting in a diminished transglutaminase activity of M2 macrophages (173). Furthermore, by activating the EP4 receptor on macrophages, PGE_2_ inhibits TNF-α and IL-12 cytokine secretion (31). The ability of lung macrophages to prevent Th2 induction in response to inhaled allergens has also been demonstrated in rats. The replacement of AM of sensitized animals by AM from naive animals completely abolished Th2 polarization by inhibition of DC allergen capture and migration to the lymph nodes (174).

Although PGE_2_ suppresses type 2 inflammation in most settings, a recent study demonstrated that PGE_2_ also has pro-inflammatory effects in murine macrophages. Mice lacking microsomal PGE_2_ synthase 1 (mPGE1) had an attenuated asthma phenotype compared to wild-type controls in response to repetitive inhalation challenges with an extract from the allergenic mold *Alternaria alternata*, which could be explained by a diminished IL-33 production by murine macrophages (175).

The pro-inflammatory prostanoid PGD_2_ binds both the DP1 and DP2 receptor on lung macrophages. DP signaling enhances migration and TNF-α secretion of both alveolar and interstitial macrophages. Furthermore, PGD_2_ also induces KC secretion from macrophages, resulting in neutrophil recruitment in the lung and this neutrophilia could be abolished by macrophage depletion (176). Interestingly, PGD_2_ synthesis by macrophages is also involved in the enhancement of airway inflammation by virus infections. Respiratory infections with RNA viruses, such as rhinovirus or respiratory syncytial virus (RSV), are associated with asthmatic exacerbations (177). To study the mechanism behind this association, Shiraishi and colleagues administered poly I:C, a synthetic dsRNA, intratracheally in OVA-sensitized rats. Those rats developed an exacerbated asthma phenotype and had elevated PGD_2_ synthesis in the lung, particularly in AMs. CRTH2-deficient animals did not exhibit a dsRNA-induced increase in eosinophil accumulation, demonstrating the necessary role for PGD_2_ in dsRNA-induced enhancement of airway inflammation (178).

RSV infection of mice deficient in 5-LOX, an enzyme required for lipoxin synthesis, resulted in stronger lung pathology compared to wildtype mice, due to a lack of alternatively activated macrophages (179, 180). Treatment with LXA_4_ partially restored this, supporting a pro-resolution role for lipoxins in viral respiratory tract infections (180).

Just as described for DCs, AMs produce leukotrienes in response to HDM-driven Dectin-2 activation. Both an inhibitor of LT production and Dectin-2 blockade could prevent the development of bronchial hyperreactivity and airway inflammation, demonstrating the required role for Dectin-2 dependent leukotriene production in the initiation of allergic airway inflammation (181).

## Summary

Allergic asthma is a chronic lung disease, driven by a prototypical Th2 response against airborne allergens. Dendritic cells (CD11b + cDC2s) are indispensable and sufficient for the development of allergic asthma, whereas macrophages have merely a tolerogenic role. Eicosanoids, leukotrienes and prostaglandins, influence key functions of these cells. However, given the diverse spectrum of eicosanoids and given the cell-type dependent expression profile of eicosanoid receptors, it is not surprising that the effects of PG/LT can be very distinct depending on the inflammatory context. A particular eicosanoid can have a pro-inflammatory effect on a certain cell type, whereas it can act anti-inflammatory on another. Furthermore, one particular cell type will be exposed to both pro-inflammatory and anti-inflammatory eicosanoids and the balance between those will determine the cellular outcome. Leukotriene receptor antagonists are already in clinical use for the treatment of asthma. In addition, given the multiple roles of prostaglandins in the pathogenesis of asthma, PG a-/antagonists may also have a promising therapeutic effect.

## Author contributions

ND wrote the first draft of the manuscript. BL edited the manuscript. Both authors contributed to manuscript revision, read and approved the submitted version.

### Conflict of interest statement

The authors declare that the research was conducted in the absence of any commercial or financial relationships that could be construed as a potential conflict of interest.

## Footnotes

^1^National Institutes of Health; National Heart, Lung, and Blood. National Asthma Education and Prevention Program Institute, Expert panel report 3: guidelines for the diagnosis and management of asthma. No. 07–4051 Available from: http://www.nhlbi.nih.gov/guidelines/asthma/asthgdln.htm 2007.
